# Independent Lineages of Highly Sulfadoxine-Resistant *Plasmodium falciparum* Haplotypes, Eastern Africa

**DOI:** 10.3201/eid2007.131720

**Published:** 2014-07

**Authors:** Steve M. Taylor, Alejandro L. Antonia, Whitney E. Harrington, Morgan M. Goheen, Victor Mwapasa, Ebbie Chaluluka, Michal Fried, Edward Kabyemela, Mwayi Madanitsa, Carole Khairallah, Linda Kalilani-Phiri, Antoinette K. Tshefu, Stephen J. Rogerson, Feiko O. ter Kuile, Patrick E. Duffy, Steven R. Meshnick

**Affiliations:** Author affiliations: Duke University Medical Center, Durham, North Carolina, USA (S.M. Taylor);; University of North Carolina, Chapel Hill, North Carolina, USA (S.M. Taylor, A.L. Antonia, M.M. Goheen, S.R. Meshnick);; Seattle Children’s Hospital/University of Washington School of Medicine, Seattle, Washington, USA (W.E. Harrington);; College of Medicine, Blantyre, Malawi (V. Mwapasa, E. Chaluluka, M. Madanitsa, L. Kalilani-Phiri);; National Institutes of Health, Bethesda, Maryland, USA (M. Fried. P.E. Duffy);; Seattle Biomedical Research Institute, Seattle (E. Kabyemela);; Liverpool School of Tropical Medicine, Liverpool, UK (C. Khairallah, F.O. ter Kuile); University of Kinshasa, Kinshasha, Democratic Republic of the Congo (A.K. Tshefu);; University of Melbourne, Melbourne, Victoria, Australia (S.J. Rogerson);; University of Amsterdam, Amsterdam, the Netherlands (F.O. ter Kuile)

**Keywords:** malaria, Plasmodium falciparum, parasites, sulfadoxine, drug resistance, lineages, genetics, haplotypes, population, eastern Africa

## Abstract

Sulfadoxine-resistant *Plasmodium falciparum* undermines malaria prevention with sulfadoxine/pyrimethamine. Parasites with a highly resistant mutant dihydropteroate synthase (*dhps*) haplotype have recently emerged in eastern Africa; they negated preventive benefits of sulfadoxine/pyrimethamine, and might exacerbate placental malaria. We explored emerging lineages of *dhps* mutant haplotypes in Malawi, the Democratic Republic of the Congo, and Tanzania by using analyses of genetic microsatellites flanking the *dhps* locus. In Malawi, a triple-mutant *dhps* SGEG (mutant amino acids are underlined) haplotype emerged in 2010 that was closely related to pre-existing double-mutant SGEA haplotypes, suggesting local origination in Malawi. When we compared mutant strains with parasites from the Democratic Republic of the Congo and Tanzania by multiple independent analyses, we found that SGEG parasites were partitioned into separate lineages by country. These findings support a model of local origination of SGEG *dhps* haplotypes, rather than geographic diffusion, and have implications for investigations of emergence and effects of parasite drug resistance.

## Abstract 2

Parasites with increased resistance to sulfadoxine might undermine malaria control measures.

Antimalarial drug resistance threatens to undermine efforts to control *Plasmodium falciparum* malaria. In sub-Saharan Africa, *P*. *falciparum* resistance to sulfadoxine/pyrimethamine (SP) is widespread, as shown by clinical treatment failures and the prevalence of molecular markers of drug resistance (*1*). Despite these findings, SP remains a major tool for malaria control when administered as a partner drug with artemisinins and as intermittent preventive therapy in infants (IPTi), children, and pregnant women (IPTp). Of these SP-based interventions, IPTi with SP is safe and effective (*2*), IPT in children receiving SP and amodiaquine has shown promise in western Africa (*3*,*4*), and IPTp-SP is used widely across sub-Saharan Africa. All 3 policies are recommended by the World Health Organization for many settings in Africa (*5*–*7*). Spread of sulfadoxine-resistant parasites will compromise the effectiveness of these programs.

IPTp-SP has been adopted most broadly; however, its efficacy appears to be decreasing in areas with increasing parasite resistance to SP (*8*,*9*). Reduced susceptibility to sulfadoxine is conferred mainly by nonsynonymous substitutions at codons 436, 437, 540, and 581 of the *P. falciparum* dihydropteroate synthase (*dhps*) gene that encodes the enzymatic target of sulfadoxine (*10*). Parasites with mutant *dhps* haplotypes are restricted to sub-Saharan Africa, and parasites with the A437G, K540E, and A581G mutations (mutant amino acids are underlined), which are known as *dhps* triple mutants (haplotype SGEG across codons 436, 437, 540, and 581), have been limited to eastern Africa. In sites in Tanzania in which the SGEG haplotype is prevalent, IPTp-SP does not appear to improve birth outcomes (*9*), and IPTi with SP is not effective (*11*).

In addition, recent evidence suggests that IPTp-SP might exacerbate placental malaria when women are infected with parasites that have the A581G mutation in *dhps* (*12*), which suggests that these parasites manifest increased pathogenicity under drug pressure. In contrast, there was no evidence of pathogenicity caused by A581G-bearing parasites in Malawi, and SP retained some efficacy in preventing illness caused by malaria during pregnancy (J. Gutman et al., unpub. data). These contrasting effects of this resistant parasite haplotype suggest that effects of the A581G mutation might vary among populations. However, if parasites from northern Tanzania dessiminate, parasites bearing the *dhps* A581G mutation could broadly undermine malaria control efforts in infants and pregnant women in Africa.

Because of these findings, molecular surveillance for this mutation is critical to assess the durability of SP for malaria prevention. Genetic studies have shown that mutations conferring resistance to chloroquine (*13*) and pyrimethamine (*14*) have arisen only a few times and then diffused across regions and continents. In contrast, resistance to sulfadoxine appears to have arisen independently in multiple locations (*15*,*16*), after originating only in Southeast Asia, followed by export to Africa (supported by global survey findings) (*17*). Efforts to prevent dissemination of the A581G mutation hinge on understanding whether the mutation arises de novo or is spread among locations.

To better understand the emergence of sulfadoxine-resistant *P*. *falciparum* in eastern Africa, we first used microsatellite genotyping to study the emergence of parasites harboring *dhps* haplotypes with the A581G mutation in a longitudinal study in Malawi during 1997–2010 (*8*). We then compared the genetic background of these triple-mutant SGEG parasites in Malawi in a cross-sectional analysis with mutant parasite haplotypes from Tanzania and the Democratic Republic of the Congo (DRC). In these 2 investigations, we hypothesized that extant SGEA haplotypes in Malawi would share a genetic lineage with recently emerged SGEG haplotypes, and that these SGEG haplotypes from Malawi would represent a distinct lineage compared with SGEG haplotypes from other settings in eastern Africa.

## Methods

### Ethics

All participants provided written or oral informed consent. Ethical approval for project activities was provided by the review boards of the Malawi Health Sciences Research Committee, the University of Malawi College of Medicine Research Ethics Committee, the Liverpool School of Tropical Medicine, Macro International, the School of Public Health of the University of Kinshasa, the International Clinical Studies Review Committee of the National Institutes of Health, the Seattle Biomedical Research Institute, the Tanzanian National Institute for Medical Research, and the University of North Carolina.

### Sample Collection

Parasites from Malawi were obtained from peripheral blood of women who delivered children at Queen Elizabeth Central Hospital in Blantyre, Malawi, during 1997–2005 (*18*). In 2010, consecutive women who delivered children at study sites near Blantyre were offered enrollment into an observational study (F.O. ter Kuile et al., unpub. data). Dried blood spots were prepared from maternal peripheral and placental blood of enrollees.

Parasites from the DRC were obtained from adults in the 2007 Demographic and Health Survey (*19*). Parasites from Tanzania were obtained from placental blood of pregnant women delivering at Muheza Designated District Hospital during 2002–2005 (*12*).

### Genotyping Procedures

For parasites from Malawi and DRC, genomic DNA was extracted from dried blood spots by using Chelex-100 or a PureLink 96 DNA Kit (Life Technologies, Grand Island, NY, USA), and *P. falciparum* was detected by using real-time PCR (*19*). These parasites were genotyped at *dhps* loci by using amplification and Sanger sequencing (*18*,*20*), and only those with pure A581G genotypes were genotyped at microsatellites. For parasites from Tanzania, the mutant alleles A437G and K540E are nearly fixed; A581G was identified by pyrosequencing (*12*). We classified parasites as having A581G if the mutant allele frequency was ≥90% within the parasitemia level of the person.

Five microsatellite loci flanking the *dhps* gene were genotyped in all isolates: −2.9 and −0.13 kB upstream, and 0.03, 0.5, and 9 kB downstream of *dhps* (*20*). PCR products of amplifications of individual loci were sized on a 310 Genetic Analyzer (Applied Biosystems, Foster City, CA, USA), and allele lengths were scored by using GeneMapper v4.1 (Applied Biosystems). In specimens with multiple peaks, the major peak was analyzed. All specimens were amplified and sized in parallel with genomic DNA from *P. falciparum* isolate 3D7 (American Type Culture Collection, Manassas, VA, USA). These controls were used to correct allele lengths to account for batch variability in fragment sizing.

### Data Analyses

We computed heterozygosity (*H_e_*) of microsatellite loci by using GenAlEx v6.5 (*21*) to quantify the degree of selection on mutant haplotypes. To assess relatedness among *dhps* haplotypes in Malawi during 1997–2010, we used GenAlEx to compute *Φ_PT_* by analysis of molecular variance (AMOVA) with 999 permutations over the whole population (*22*) and the Nei genetic distance (*23*) among *dhps* haplotypes and years based on microsatellite profiles. We inputted *Φ_PT_* values computed by AMOVA into a principal coordinates analysis (PCoA) in GenAlEx.

We further characterized these relationships with a network analysis. To characterize these relationships, we assigned unique haplotypes based on microsatellite profiles for the 91 isolates for which we had successfully genotyped all microsatellite loci. These unique haplotypes were inputted into NETWORK v4.6.1.1 (*24**,**25*), and weights were assigned to each locus in inverse proportion to the *H_e_*, of the locus, as calculated above.

In cross-sectional analysis of parasite populations from eastern Africa defined by location and *dhps* haplotype, we first used GenAlEx to compute pairwise linear genetic distances and *Φ_PT_* (by using AMOVA with 999 permutations over the full population) and then used SPAGeDi v1.4 (*26*) to compute pairwise *R_ST_* (by using jackknifing with 1,000 permutations). We inputted pairwise tri-distance matrices of linear genetic distance, *Φ_PT_*, and *R_ST_* into separate PCoAs in GenAlEx. For testing of statistical significance, we considered a p value of 0.05 as sufficient to reject the null hypothesis and used the Bonferroni correction when computing multiple comparisons.

We constructed a neighbor-joining (NJ) network to estimate a phylogeny of *dhps* haplotypes circulating in eastern Africa. To construct this network, we first computed pairwise linear genetic distances among all 193 isolates in GenAlEx; this distance matrix was used to compute an unrooted NJ tree in PHYLIP v3.67 (*27**,**28*), which was computed agnostic to *dhps* haplotype and geographic location and rendered in R v3.0.1. Missing alleles precluded computation of a median-joining network with NETWORK (Technical Appendix Table 1).

We investigated population structure of the *dhps* haplotypes from these 193 isolates by using STRUCTURE v2.3.4, a clustering algorithm designed to infer and assign individuals to subpopulations (*29*). Although it was not specifically designed to identify population structure based on linked loci, we used STRUCTURE to test our a priori hypothesis of distinct subpopulations based on *dhps* haplotype and location (*30*). We performed 3 analyses: first, of all 193 parasites of all *dhps* haplotypes; second, of 116 parasites with any mutant *dhps* haplotype; and third, of 32 parasites with only the triple-mutant SGEG *dhps* haplotype. We performed 5 simulations each at values of *K*-estimated populations from 1 to 20, and estimated the true *K* a posteriori by using estimations in STRUCTURE, as well as using the method of Evanno et al. (*31*)

## Results

### Longitudinal Analyses of Parasites in Malawi

We first tested 114 *P. falciparum* isolates from Malawi collected during 1997–2010. We identified 25 wild-type SAKA parasites, 1 single-mutant SGKA, 68 double-mutant SGEA, 10 triple-mutant SGEG, and 10 with other *dhps* haplotypes, including AAKA, AGEA, AGKA, and SAEA. Among major haplotypes, we observed reductions in microsatellite allele mean heterozygosity (*H_e_)* in parasites having double-mutant SGEA (*H_e_* 0.454, SE 0.076) and triple-mutant SGEG *dhps* haplotypes (*H_e_* 0.485, SE 0.134) compared with those from wild-type parasites (*H_e_* 0.798, SE 0.064). These findings are consistent with positive selection on mutant haplotypes, presumably caused by sulfadoxine pressure.

Given the recent emergence of triple-mutant SGEG haplotype in 2010, we investigated its relationship with the double-mutant SGEA haplotype that had become fixed in this population by 2005 (*32*). To quantify genetic relatedness among years and major *dhps* haplotypes, we first computed pairwise *Φ_PT_* values and Nei genetic distance among major *dhps* haplotypes binned by year. In these analyses, SGEA haplotypes were closely related to each other during 1997–2005 (*Φ_PT_* values 0.008–0.065, Nei value 0.016–0.073) and closely related to the SGEG haplotype that emerged by 2010 (*Φ_PT_* 0.082, Nei value 0.049) (Table). We inputted *Φ_PT_* estimates into a PCoA to better visualize divergence among haplotypes by year. In this analysis, coordinates 1 and 2 explained 96.3% of the variance; SGEA haplotypes from all years clustered with SGEG haplotypes from 2010, which suggested a shared lineage in Malawi of mutant *dhps* haplotypes during 1997–2010 (Technical Appendix Figure 1).

**Table:** Pairwise Φ*_PT_* values and Nei genetic distances among major *Plasmodium falciparum* *dhps* haplotypes by year, Malawi*

Haplotype, year	SAKA, 1997–1999	SAKA, 2000–2003	S**GE**A, 1997–1999	S**GE**A, 2000–2003	S**GE**A, 2004–2005	S**GE**A, 2010	S**GEG**, 2010
SAKA, 1997–1999		0.251	0.470	0.693	0.818	1.095	1.044
SAKA, 2000–2003	0		0.362	0.264	0.200	0.350	0.162
S**GE**A, 1997–1999	**0.249**	**0.331**		0.02	0.073	0.429	0.246
S**GE**A, 2000–2003	**0.295**	**0.347**	0.008		0.016	0.302	0.096
S**GE**A, 2004–2005	**0.318**	**0.404**	0.065	0.030		0.300	0.049
S**GE**A, 2010	**0.299**	**0.380**	**0.298**	**0.243**	**0.237**		0.031
S**GEG**, 2010	**0.243**	0.299	**0.202**	**0.155**	0.082	0.015	

*Values for *dhps* haplotypes are defined by amino acids at codons 436, 437, 540, and 581. Mutant amino acids are underlined and in bold. Pairwise *Φ_PT_* values are shown below the diagonal; values in bold have a p value <0.05 (after Bonferroni correction for multiple comparisons) based on 999 permutations. Nei unbiased genetic distances are shown above the diagonal.

**Figure 1:**
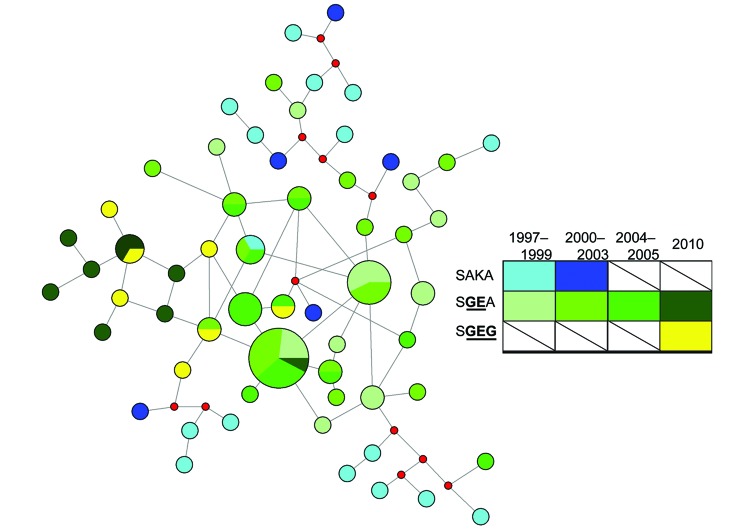
Genetic relatedness of *Plasmodium falciparum* dihydropteroate synthase (*dhps*) haplotypes from Malawi over time based on median-joining network of microsatellite profiles. Median-joining network was calculated based on microsatellite profiles for 91 parasites with full genotype data. Colors indicate year and *dhps* haplotype, nodes are proportional to the number of parasites with that microsatellite profile, red nodes are hypothetical profiles inserted by the program to calculate a parsimonious network, and branch lengths are arbitrary. Values were computed in NETWORK v4.6.1.1. (*24**,**25*). The *dhps* haplotypes are defined by amino acids at codons 436, 437, 540, and 581. Mutant amino acids are underlined and in bold. SAKA, wild-type; SGEA and SGEG, mutants.

We further investigated this finding by using network analysis. To perform this analysis, we constructed a median-joining network of wild-type and mutant haplotypes by year based on microsatellite profiles (Figure 1). In this analysis, we observed clustering of triple-mutant SGEG haplotypes from 2010 in a network of double-mutant SGEA haplotypes, as well as substantial sharing of microsatellite profiles among SGEA parasites from different years and with SGEG parasites. These 2 observations suggest a shared lineage of evolved mutant *dhps* haplotypes in Malawi.

### Cross-sectional Analyses of Parasite Haplotypes for Eastern Africa

Clinical consequences of infections with parasites bearing the *dhps* A581G mutation appear to vary among study sites in Africa. In Tanzania, these parasites have been associated with exacerbation of placental inflammation in women who received IPTp (*12*) and failure of IPTp-SP to prevent low birthweight of infants (*33*). In Malawi, these phenomena have not yet been observed. Because of these differing effects, we speculated that haplotypes bearing the A581G mutation may also differ among sites.

We conducted a cross-sectional analysis of parasites from 2 additional cohorts: 1) adults sampled in 2007 from the eastern DRC (*19*), and 2) pregnant women who gave birth and were enrolled during 2002–2005 in Muheza, Tanzania (*12*). In total, we compared the genetic relationships among 193 parasites grouped into 7 parasite populations: wild-type (SAKA) isolates from the DRC (n = 53) and Malawi (n = 24), those bearing double-mutant (SGEA) haplotypes from the DRC (n = 17) and Malawi (n = 67), and those bearing triple-mutant (SGEG) haplotypes from the DRC (n = 5), Malawi (n = 10), and Tanzania (n = 17). Fragment lengths are shown in Technical Appendix Table 1.

We quantified divergence of these 7 populations based on microsatellite allele lengths by using 3 population genetic metrics: linear genetic distance, *Φ_PT_,* and *R_ST_*. Linear genetic distance is a simple Euclidean genetic distance metric, and *Φ_PT_,* and *R_ST_* are variations of Wright F-statistics that quantify divergence and estimate its variance (*22**,**34*). Among SGEG parasites from Malawi and Tanzania, *Φ_PT_* (0.420, p = 0.001) and *R_ST_* (0.436, p<0.0001) indicated significant divergence after Bonferroni correction for multiple comparisons (Technical Appendix Table 2); *Φ_PT_* and *R_ST_* for other pairwise comparisons among SGEG parasites from Malawi, the DRC, and Tanzania were not significant but suggested similar divergence (values >0.420).

**Figure 2:**
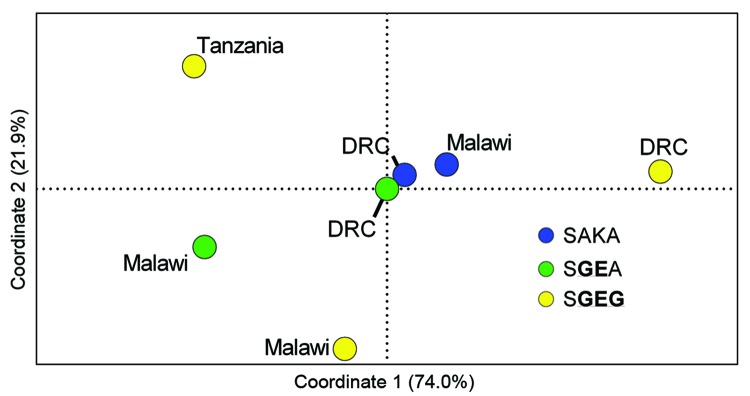
Principal coordinates analyses of wild-type (SAKA) and mutant (S**GE**A and S**GEG**) *Plasmodium falciparum* dihydropteroate synthase (*dhps*) halotypes from eastern Africa based on analysis of variance (*R_ST_*). Pairwise *R_ST_* values were computed with SPAGeDi (*26*) for microsatellite profiles of 7 populations of parasites defined by *dhps* haplotype and location: SAKA parasites from Malawi (n = 24) and the Democratic Republic of the Congo (DRC) (n = 53) (blue dots); SGEA parasites from Malawi (n = 67) and DRC (n = 17) (green dots); and SGEG parasites from DRC (n = 5), Malawi (n = 10), and Tanzania (n = 17) (yellow dots). These pairwise values were inputted into principal coordinates analyses in GenAlEx (*21*), in which coordinates 1 and 2 cumulatively accounted for 96% of the variance. The *dhps* haplotypes are defined by amino acids at codons 436, 437, 540, and 581. Mutant amino acids are underlined and in bold.

We visualized the output of each of these metrics with separate PCoAs (Figure 2; Technical Appendix Figure 2). In the PCoAs of genetic distance, *Φ_PT_,* and *R_ST_*, the first 2 coordinates accounted for 89%, 94.3%, and 96% of variance in values, respectively. In each PCoA plot, SGEG parasites from Malawi, the DRC, and Tanzania were consistently distant from each other in the 2 plotted dimensions, and other relationships among populations were variable. These analyses suggested divergence of SGEG haplotypes in Malawi, Tanzania, and the DRC.

**Figure 3:**
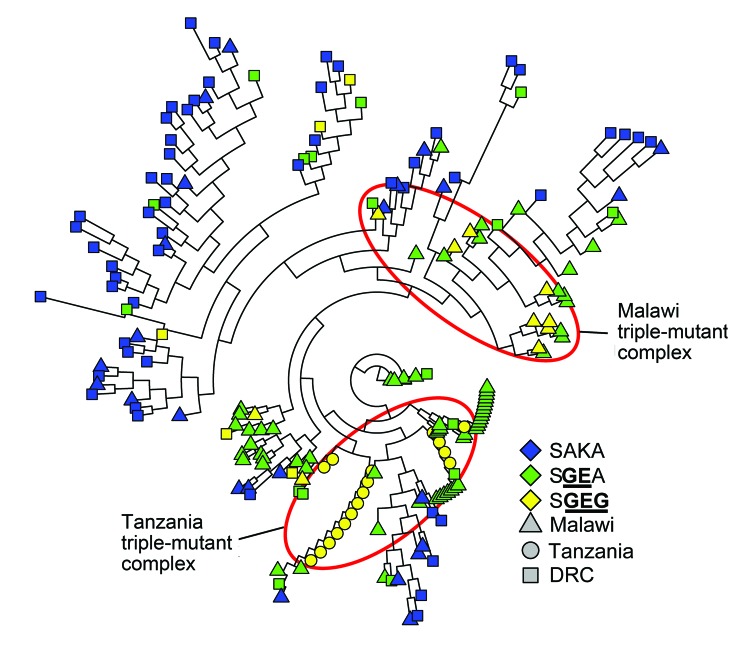
Neighbor-joining network of *Plasmodium falciparum* wild-type (SAKA) and mutant (SGEA and SGEG) dihydropteroate synthase (*dhps*) haplotypes, eastern Africa. Pairwise linear genetic distances among 193 parasite isolates were computed in GenAlEx (*21*) inputted into PHYLIP v3.67 (*27**,**28*) to calculate an unrooted neighbor-joining tree, and rendered in R v3.0.1 (http://www.r-project.org/) by using the ape package. For visualization, branch lengths were uniformly lengthened if not equal to 0. The *dhps* haplotypes are defined by amino acids at codons 436, 437, 540, and 581. Mutant amino acids are underlined and in bold. DRC, the Democratic Republic of the Congo.

To further investigate this apparent divergence of SGEG haplotypes, we computed an NJ network based on pairwise linear genetic distances among all 193 isolates. This phylogenetic analysis, which was computed agnostic to country and *dhps* haplotype, clustered all 17 SGEG parasites from Tanzania distinctly from 8 of the 10 SGEG parasites from Malawi; haplotypes in these clusters were also distinct from 3 of the 5 SGEG parasites from the DRC (Figure 3). A large number of SGEA parasites from Malawi also clustered closely with SGEG parasites from Tanzania, which suggested some shared lineage. This inferred phylogeny further suggests that triple-mutant *dhps* haplotypes from Tanzania and Malawi bearing the A581G substitution have arisen independently.

**Figure 4:**
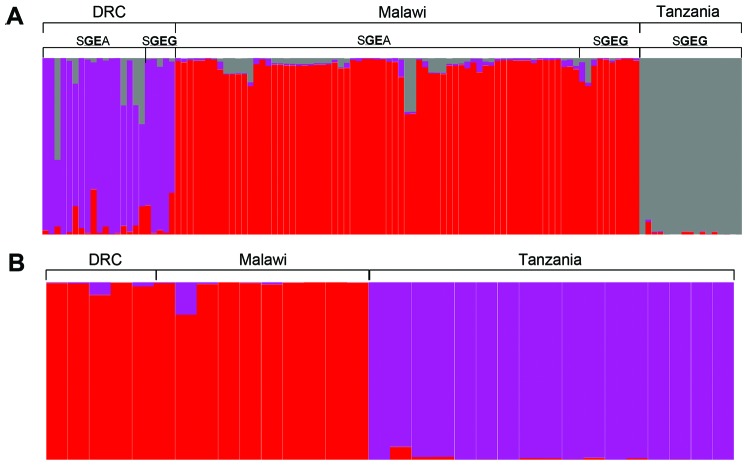
Predicted population structure of *Plasmodium falciparum* parasites from eastern Africa among those with A) double-mutant (S**GE**A) or triple-mutant (S**GEG**) dihydropteroate synthase (*dhps*) haplotypes and B) those with only the triple-mutant S**GEG** *dhps* haplotype. Estimations of number of populations and assignments of individual parasites to clusters were computed with STRUCTURE (*29**,**30*) by using a burn-in period of 10,000 and 10,000 iterations and models that assume no admixture and which use prior information related to location because of sparse data and likely subtle structure. Each vertical bar represents an individual parasite, and the color of each bar indicates the likelihood of membership in the subpopulation indicated by the color. The *dhps* haplotypes are defined by amino acids at codons 436, 437, 540, and 581. Mutant amino acids are underlined and in bold. A) Analysis of 116 parasites from Malawi, the Democratic Republic of the Congo (DRC), and Tanzania with either S**GE**A or S**GEG** *dhps* haplotypes. The posterior probability was 99.9% of the existence of 3 population clusters that are indicated by the color. B) A similar analysis restricted to 32 parasites with only the S**GEG** *dhps* haplotype from Malawi, DRC, and Tanzania. The division into 2 populations indicated by the color was supported by a posterior probability of 41%, which is higher than for those of other a priori population sizes tested.

To further investigate this partition of *dhps* lineages, we performed an analysis of population structure. The first analysis of all 193 wild-type and mutant parasites partitioned only *dhps* haplotypes into mutant and wild-type, irrespective of geographic location. When restricted to the 116 parasites with mutant *dhps* haplotypes, the program partitioned the dataset into 3 clusters with 99.9% posterior probability; these 3 clusters directly corresponded to geographic location but were irrespective of *dhps* haplotype, which suggested separate lineages of mutant *dhps* haplotypes in Malawi, the DRC, and Tanzania (Figure 4, panel A). In an analysis restricted to the 32 parasites bearing the SGEG *dhps* haplotype, 2 population clusters were identified with the highest posterior probability. All SGEG parasites from Tanzania were assigned to 1 cluster, and SGEG parasites from Malawi and the DRC were assigned to a second cluster (Figure 4, panel B). These analyses further suggest that lineages of triple-mutant *dhps* haplotypes bearing the A581G mutation from Malawi and Tanzania are divergent.

## Discussion

In these longitudinal and cross-sectional analyses of *P*. *falciparum* haplotypes from eastern Africa that were associated with high-level parasite sulfadoxine resistance, we demonstrated emergence of a distinct lineage of triple-mutant *dhps* haplotypes in Malawi. In comparative analyses, these *dhps* triple-mutant SGEG haplotypes from Malawi were strongly divergent from haplotypes collected in northern Tanzania, which suggested independent emergence of this drug-resistant haplotype in these 2 settings in eastern Africa. This parasite haplotype appears to undermine efficacy of SP in preventing *P*. *falciparum* infection in infants and pregnant women (*9*,*11*); its spread could undermine current and nascent malaria control programs that are predicated on SP efficacy. To guide malaria control policies that use SP, surveillance of molecular markers of *P. falciparum* drug resistance need to be complemented by clinical studies of effects of these parasite mutations on maternal and infant birth outcomes.

Our data indicate that triple-mutant SGEG *dhps* haplotypes from Malawi, Tanzania, and the DRC represent distinct lineages. The circumscribed regional distribution is well described for single-mutant and double-mutant *dhps* haplotypes across sub-Saharan Africa (*15**,**20*), but our study describes this phenomenon on a subregional scale with the emerging, more highly resistant triple-mutant SGEG haplotype. In our analyses, support for this genetic divergence derives from population genetic metrics, including genetic distance and F-statistics (Technical Appendix Table 2), PCoA (Figure 2; Technical Appendix Figure 2), unsupervised haplotype phylogeny (Figure 3), and inferred population structure (Figure 4).

Parasites from Malawi and Tanzania were most consistently partitioned into distinct SGEG lineages and showed statistically significant divergence by *Φ_PT_* (0.42, p = 0.001) and *R_ST_* (0.436, p<0.0001) (Technical Appendix Table 2), visually apparent partitioning in an NJ network (Figure 3) and in PCoA (Figure 2), and assignment to separate subpopulations in a probabilistic clustering algorithm (Figure 4). In a previous study, double-mutant SGEA haplotypes in eastern Africa (including Malawi, Tanzania, and eastern DRC) shared a common lineage (*15*). Our study supports this finding for parasites from Malawi and the DRC, with closely related SGEA parasites identified by *Φ_PT_* (0.017, p = 0.228) and *R_ST_* (0.0164, p = 0.4306) (Technical Appendix Table 2). Our data suggest that despite this shared double-mutant *dhps* haplotype, the additional A581G mutation has arisen independently in different locations in, at least, Malawi and Tanzania.

Recent studies of pregnant women have implicated parasites bearing the SGEG *dhps* haplotype with undermining the efficacy of IPTp-SP (J. Gutman et al., unpub. data, *33*) and possibly exacerbating placental infection in women who received IPTp-SP (*12*). Despite these findings, results of various studies are inconsistent regarding the effect of the SGEG haplotype on IPTp-SP. On a population level, IPTp-SP appears to retain some efficacy in preventing low birthweight in infants in studies in Malawi, where the SGEG haplotype is just emerging (*35*), but not in northern Tanzania, where SGEG parasites are more prevalent (*9*). On an individual level, IPTp-SP is less effective at preventing peripheral parasitemia at delivery caused by parasites bearing the SGEG haplotype in Malawi (J. Gutman et al., unpub. data), but IPTp-SP was implicated as exacerbating placental parasite density and inflammation in the presence of SGEG parasites in Tanzania (*12*). These inconsistencies in ecologic studies may derive from different frequencies of the A581G mutation in parasite populations in the 2 settings.

A second explanation for the differences is that these effects might be derived from additional mutations that are associated with genotyped loci caused either by different genetic backgrounds or linkage disequilibrium. Such mutations may be hypothesized to exist in the *dhps* gene itself, in loci which mediate DHPS expression and thereby parasite fitness in the presence of drug, or in heretofore undescribed mediators of pathogenesis. In areas in which parasites bearing the SGEG haplotypes are emerging, studies of parasite genomics and phenotypes can further investigate these possibilities.

Our results suggest atypical spread in Africa of parasite haplotypes conferring sulfadoxine resistance: haplotypes that confer resistance to chloroquine (*13*) and pyrimethamine (*14*) appear to have limited origins and are believed to have spread across Africa largely by gene flow. Similarly, a previous global survey of *dhps* haplotypes suggested that resistant lineages originated in Southeast Asia and migrated to Africa (*17*), although this study did not include any parasites from Africa with the SGEG *dhp*s haplotype. In contrast, our data support the local origination and emergence of this highly resistant haplotype, similar to rare resistant *dhfr* haplotypes (*36*). This phenomenon for *dhps* has been most clearly described on a fine scale in Southeast Asia (*16*), and our data support a similar process in eastern Africa.

This model is further supported by our longitudinal sampling and testing of parasite specimens in Malawi during 1997–2010. During this period, SGEG haplotypes arose after SGEA haplotypes had achieved near fixation. This observation could have been caused by importation of a new parasite population bearing the SGEG *dhps* haplotype or by A581G mutation in the existing parasite population. Our data suggest that the mutation accounts for appearance of the SGEG haplotype because of the close relationship among mutant haplotypes both computationally by *Φ_PT_* and visually in a median-joining network (Figure 1), which suggests that SGEA haplotypes gave rise to SGEG haplotypes in Malawi.

What are the implications of our findings for IPTp-SP? IPTp-SP is still used extensively in settings in eastern and southern Africa that have prevalent parasite resistance. Failures of IPTp-SP have prompted investigations to define the prevalence of the A581G mutation (*37*) and to more closely investigate its potential for modifying the beneficial effect of IPTp-SP. Our results have 2 practical implications for these investigations. First, these data suggest that the SGEG haplotype may arise where a SGEA haplotype is circulating without requiring the importation of a new mutant haplotype. Therefore, even in settings without major migration of parasites, continued use of sulfadoxine or other sulfonamides may promote emergence of this haplotype. Molecular surveillance is critical for detecting this step. Second, given the differing effects of parasites bearing the SGEG haplotype on IPTp-SP among settings, it would appear prudent to supplement molecular surveillance for the *dhps* A581G mutation in areas where it is present by studies of its effects on pregnant women and their infants and other clinical outcomes.

Our study has several limitations. First, the number of available parasites bearing the SGEG haplotype is limited. These parasites have emerged only recently in Africa, precluding a more widespread analysis. Second, we did not have SGEA parasites from Tanzania against which to compare microsatellite profiles of the SGEG parasites. Third, these SGEG parasites were collected in different clinical studies in different years; the 2 principal populations of parasites were collected from women giving birth in Malawi and Tanzania during 2002–2010 and are therefore quite similar.

These analyses of malaria parasites in eastern Africa support a model of local origination and propagation of triple-mutant SGEG *P. falciparum dhps* haplotypes that confer high levels of resistance to sulfadoxine. Recent evidence indicates that these haplotypes abrogate the efficacy of SP use to prevent malaria among pregnant women and their infants in eastern Africa. Integrated clinical and molecular surveillance for these mutations in parasite populations is critical to assess the durability of prevention programs that use SP. These efforts should be complemented with ongoing investigations of more effective methods to protect vulnerable populations from malaria.

Technical Appendix Additional population genetic indices and fragment lengths of microsatellite loci for Independent lineages of highly sulfadoxine-resistant *Plasmodium falciparum* haplotypes, eastern Africa.
