# Supplementary material for: Independent Lineages of Highly Sulfadoxine-Resistant Plasmodium falciparum Haplotypes, Eastern Africa
Source: Emerg Infect Dis. 2014 Jul;20(7):1140–8. doi: 10.3201/eid2007.131720 (PMC4073871; doi:10.3201/eid2007.131720)
Supplement: Technical Appendix — Additional population genetic indices and fragment lengths of microsatellite loci for Independent lineages of highly sulfadoxine-resistant Plasmodium falciparum haplotypes, eastern Africa. [file 13-1720-Techapp-s1.pdf]

Article DOI: http://dx.doi.org/10.3201/eid2007.131720

Independent Lineages of Highly Sulfadoxine-Resistant *Plasmodium falciparum* Haplotypes, Eastern Africa

Technical Appendix

Additional population genetic indices and fragment lengths of microsatellite loci for Independent lineages of highly sulfadoxine-resistant *Plasmodium falciparum* haplotypes, eastern Africa.

Technical Appendix Table 1. Lengths of microsatellite loci flanking the *Plasmodium falciparum dhps* gene, by country of origin in eastern Africa and *dhps* haplotype*

		Loci				
Country	Haplotype†	–2.9	–0.13	0.03	0.5	9
DRC	SGEA	194	134	124	147	108
DRC	SGEA	182	0	122	0	0
DRC	SGEA	194	136	140	145	106
DRC	SGEA	202	142	128	147	106
DRC	SGEA	194	0	126	0	106
DRC	SGEA	194	136	142	147	106
DRC	SGEA	182	136	140	147	110
DRC	SGEA	194	136	140	147	114
DRC	SGEA	194	136	142	145	104
DRC	SGEA	194	136	128	147	108
DRC	SGEA	194	136	132	147	106
DRC	SGEA	192	136	140	147	124
DRC	SGEA	194	136	128	147	108
DRC	SGEA	194	136	140	147	106
DRC	SGEA	194	136	204	147	106
DRC	SGEA	194	136	140	147	106
DRC	SGEA	194	136	142	145	106
DRC	SGEG	194	136	142	147	108
DRC	SGEG	0	0	126	0	0
DRC	SGEG	194	136	122	145	106
DRC	SGEG	194	0	126	147	108
DRC	SGEG	194	136	142	145	108
DRC	SGKA	182	142	128	147	108
DRC	SGKA	182	142	128	147	106
DRC	SGKA	196	134	126	147	98
DRC	SGKA	182	142	126	147	108
DRC	SGKA	196	136	126	145	126
DRC	SGKA	196	142	126	147	106
DRC	SGKA	182	142	126	147	106
DRC	SGKA	182	142	154	145	106
DRC	SGKA	182	142	126	145	106
DRC	SGKA	182	142	128	145	106
DRC	SGKA	182	142	126	147	110
DRC	SGKA	182	142	126	145	118
DRC	SGKA	182	142	128	145	108
DRC	SGKA	0	142	142	0	106
DRC	SGKA	182	142	128	145	106
DRC	SGKA	182	134	128	0	122
DRC	SGKA	182	142	128	145	110
DRC	SGKA	182	142	128	145	106
DRC	SGKA	0	142	0	0	108

		Loci				
Country	Haplotype†	–2.9	–0.13	0.03	0.5	9
DRC	SGKA	182	142	128	147	110
DRC	SGKA	182	136	128	0	106
DRC	SGKA	182	134	126	143	130
DRC	SGKA	182	142	126	145	106
DRC	SGKA	182	142	126	145	108
DRC	SGKA	196	136	128	147	106
DRC	SGKA	182	142	126	147	102
DRC	SGKA	182	0	126	145	106
DRC	SGKA	182	134	128	145	104
DRC	SGKA	182	142	128	145	106
DRC	SGKA	182	142	128	145	108
DRC	SGKA	182	142	126	147	106
DRC	SGKA	182	138	126	147	106
DRC	SGKA	182	142	128	145	108
DRC	SGKA	182	142	128	147	106
DRC	SGKA	182	136	128	145	106
DRC	SGKA	182	142	126	147	106
DRC	SGKA	182	142	128	145	106
DRC	SGKA	182	136	126	147	130
DRC	SGKA	0	142	128	143	106
DRC	SGKA	182	142	128	0	106
DRC	SGKA	182	142	126	147	106
Malawi	SGEA	194	134	142	145	102
Malawi	SGEA	194	136	142	147	102
Malawi	SGEA	194	136	142	145	104
Malawi	SGEA	194	136	142	147	104
Malawi	SGEA	194	136	142	147	104
Malawi	SGEA	196	136	142	149	104
Malawi	SGEA	194	134	142	145	106
Malawi	SGEA	192	136	142	145	106
Malawi	SGEA	194	136	142	145	106
Malawi	SGEA	194	136	142	145	106
Malawi	SGEA	194	136	142	145	106
Malawi	SGEA	194	136	142	145	106
Malawi	SGEA	194	136	140	147	106
Malawi	SGEA	196	138	140	147	106
Malawi	SGEA	194	134	142	147	106
Malawi	SGEA	182	136	142	147	106
Malawi	SGEA	194	136	142	147	106
Malawi	SGEA	194	136	142	147	106
Malawi	SGEA	194	136	142	147	106
Malawi	SGEA	194	136	142	147	106
Malawi	SGEA	194	136	142	147	106
Malawi	SGEA	194	136	142	147	106
Malawi	SGEA	194	136	142	147	106
Malawi	SGEA	194	136	142	147	106
Malawi	SGEA	194	136	142	147	106
Malawi	SGEA	194	136	142	147	106
Malawi	SGEA	194	136	140	149	106
Malawi	SGEA	194	136	142	0	106
Malawi	SGEA	194	136	144	0	106
Malawi	SGEA	194	136	142	147	108
Malawi	SGEA	194	136	140	149	108
Malawi	SGEA	196	136	142	149	108
Malawi	SGEA	196	136	142	149	108
Malawi	SGEA	194	134	142	145	112
Malawi	SGEA	194	136	142	145	112
Malawi	SGEA	194	136	142	145	116
Malawi	SGEA	196	138	142	149	116
Malawi	SGEA	208	134	140	145	106
Malawi	SGEA	194	136	140	145	106
Malawi	SGEA	194	136	140	145	106
Malawi	SGEA	194	134	142	145	106
Malawi	SGEA	186	136	142	145	106
Malawi	SGEA	194	136	142	145	106
Malawi	SGEA	194	136	142	145	106
Malawi	SGEA	194	136	142	145	106
Malawi	SGEA	194	136	142	147	106

		Loci				
Country	Haplotype†	–2.9	–0.13	0.03	0.5	9
Malawi	SGEA	194	136	142	147	106
Malawi	SGEA	194	136	142	147	106
Malawi	SGEA	194	136	144	147	106
Malawi	SGEA	194	134	140	145	108
Malawi	SGEA	182	136	140	145	108
Malawi	SGEA	194	136	140	145	108
Malawi	SGEA	194	136	140	145	108
Malawi	SGEA	196	136	140	145	108
Malawi	SGEA	194	136	142	145	108
Malawi	SGEA	194	136	142	145	108
Malawi	SGEA	194	136	142	147	108
Malawi	SGEA	194	136	142	147	108
Malawi	SGEA	194	136	142	147	108
Malawi	SGEA	194	0	140	145	110
Malawi	SGEA	194	134	142	145	110
Malawi	SGEA	194	136	142	147	110
Malawi	SGEA	194	0	142	147	110
Malawi	SGEA	194	134	142	145	112
Malawi	SGEA	194	136	142	145	120
Malawi	SGEA	194	136	142	145	126
Malawi	SGEA	194	0	0	0	0
Malawi	SGEG	194	136	132	147	106
Malawi	SGEG	194	136	140	147	106
Malawi	SGEG	194	136	140	147	108
Malawi	SGEG	196	136	142	149	110
Malawi	SGEG	194	136	142	0	112
Malawi	SGEG	0	136	142	147	0
Malawi	SGEG	196	136	142	0	0
Malawi	SGEG	196	136	142	149	106
Malawi	SGEG	196	136	142	149	108
Malawi	SGEG	194	136	142	147	110
Tanzania	SGEG	194	136	140	145	106
Tanzania	SGEG	194	136	142	147	106
Tanzania	SGEG	194	136	140	147	106
Tanzania	SGEG	194	136	140	147	106
Tanzania	SGEG	194	136	140	145	106
Tanzania	SGEG	194	136	140	145	106
Tanzania	SGEG	194	136	140	145	106
Tanzania	SGEG	194	136	142	145	106
Tanzania	SGEG	194	136	142	145	106
Tanzania	SGEG	194	136	140	145	106
Tanzania	SGEG	194	136	142	145	106
Tanzania	SGEG	194	136	140	145	106
Tanzania	SGEG	194	136	142	145	106
Tanzania	SGEG	194	136	140	145	106
Tanzania	SGEG	194	136	140	145	106
Tanzania	SGEG	194	136	140	145	106
Tanzania	SGEG	194	136	140	145	106
Malawi	SAKA	198	134	146	143	104
Malawi	SAKA	190	136	140	143	106
Malawi	SAKA	198	134	134	145	106
Malawi	SAKA	194	136	140	145	106
Malawi	SAKA	198	134	146	145	106
Malawi	SAKA	0	134	138	149	106
Malawi	SAKA	192	134	130	141	108
Malawi	SAKA	186	136	138	141	108
Malawi	SAKA	194	134	128	143	108
Malawi	SAKA	182	136	130	145	108
Malawi	SAKA	184	134	138	145	108
Malawi	SAKA	182	136	118	141	110
Malawi	SAKA	184	134	126	143	110
Malawi	SAKA	194	136	144	145	110
Malawi	SAKA	194	134	132	149	110
Malawi	SAKA	206	134	138	145	112
Malawi	SAKA	186	136	142	145	112
Malawi	SAKA	182	134	138	151	112
Malawi	SAKA	186	136	130	145	114
Malawi	SAKA	190	136	132	0	114

		Loci				
Country	Haplotype†	–2.9	–0.13	0.03	0.5	9
Malawi	SAKA	190	134	132	141	116
Malawi	SAKA	0	142	0	0	116
Malawi	SAKA	198	132	136	143	120
Malawi	SAKA	196	134	138	145	138
DRC	SAKA	188	134	128	141	104
DRC	SAKA	182	136	122	143	104
DRC	SAKA	198	134	132	141	108
DRC	SAKA	188	134	128	143	116
DRC	SAKA	194	134	132	141	110
DRC	SAKA	194	134	128	147	108
DRC	SAKA	182	134	136	0	110
DRC	SAKA	194	134	128	143	110
DRC	SAKA	208	134	126	149	114
DRC	SAKA	0	138	138	145	106
DRC	SAKA	182	136	116	143	108
DRC	SAKA	198	136	128	141	112
DRC	SAKA	190	134	138	145	108
DRC	SAKA	188	134	134	133	120
DRC	SAKA	196	134	126	145	108
DRC	SAKA	182	0	136	0	120
DRC	SAKA	182	142	126	143	106
DRC	SAKA	182	134	124	145	114
DRC	SAKA	196	136	126	147	106
DRC	SAKA	182	134	126	143	110
DRC	SAKA	198	138	148	143	110
DRC	SAKA	0	136	128	139	126
DRC	SAKA	182	134	130	141	102
DRC	SAKA	204	138	136	145	110
DRC	SAKA	182	138	130	145	104
DRC	SAKA	196	142	124	143	112
DRC	SAKA	190	134	126	143	104
DRC	SAKA	196	134	134	141	132
DRC	SAKA	198	134	128	143	106
DRC	SAKA	182	142	128	147	106
DRC	SAKA	184	136	142	147	118
DRC	SAKA	0	0	128	145	108
DRC	SAKA	192	136	136	145	116
DRC	SAKA	198	134	138	0	110
DRC	SAKA	182	134	128	149	106
DRC	SAKA	190	136	204	149	106
DRC	SAKA	190	136	122	147	108
DRC	SAKA	194	134	122	145	108
DRC	SAKA	190	134	204	0	110
DRC	SAKA	186	134	128	145	112
DRC	SAKA	190	134	134	141	110
DRC	SAKA	0	0	122	143	118
DRC	SAKA	188	134	140	145	100
DRC	SAKA	182	134	136	145	108
DRC	SAKA	182	136	0	143	102
DRC	SAKA	210	134	136	145	104
DRC	SAKA	186	136	132	145	110
DRC	SAKA	182	134	104	143	108
DRC	SAKA	0	0	126	151	108
DRC	SAKA	188	134	132	145	110
DRC	SAKA	194	134	120	143	104
DRC	SAKA	196	136	130	87	104
DRC	SAKA	198	134	128	143	108

*Fragment lengths have been corrected by the size of alleles in *P. falciparum* isolate 3d7 to account for batch variability. Major peaks are included. Zeros indicate allele is missing. *dhps*, dihydropteroate synthase; DRC, the Democratic Republic of the Congo..

†*dhps* haplotypes are defined by amino acids at codons 436, 437, 540, and 581.

Technical Appendix Table 2. Pairwise genetic distances, Φ_{PT}, and R_{ST} values for *Plasmodium falciparum* dihydropteroate synthase haplotypes, eastern Africa*

First population	Second population	Genetic distance	Φ_{PT}	p value	R_{ST}	p value
Malawi	DRC	28.738	0	0.409	0	0.8322
DRC	Malawi	18.128	0.017	0.228	0.0164	0.4306
DRC	Malawi	13.558	0.470	0.006	0.469	0.014
DRC	Tanzania	12.118	0.659	0.009	0.660	0.017
Malawi	Tanzania	7.190	0.420	0.001	0.436	<0.0001
DRC	DRC	17.820	0	0.333	0	0.9041
DRC	Malawi	17.305	0	0.391	0	0.2627
DRC	Tanzania	15.772	0	0.400	0	0.5764
DRC	Malawi	26.766	0.010	0.369	0.009	0.6783
DRC	DRC	31.951	0.037	0.124	0.036	0.2348
DRC	Malawi	14.043	0.613	0.001	0.606	<0.0001
DRC	Malawi	21.351	0.020	0.340	0.001	0.7802
DRC	DRC	24.939	0	0.306	0	0.4885
Malawi	Malawi	7.457	0.039	0.163	0.033	0.4006
Malawi	Tanzania	7.173	0.060	0.041	0.060	0.0849
Malawi	Malawi	5.772	0.347	0.001	0.350	<0.0001
Malawi	DRC	30.636	0.211	0.001	0.213	<0.0001
Malawi	Malawi	20.680	0.172	0.005	0.165	0.021
Malawi	DRC	29.181	0.089	0.052	0.085	0.1109
Tanzania	Malawi	20.186	0.240	0.001	0.244	<0.0001
Tanzania	DRC	28.883	0.104	0.021	0.105	0.035

*Linear genetic distance and Φ_{PT} were computed with GenAlEx (http://biology.anu.edu.au/GenAlEx/Welcome.html) by using analysis of molecular variance and 999 permutations. R_{ST} was computed with SPAGeDi (http://ebe.ulb.ac.be/ebe/SPAGeDi.html) by using jackknifing and 1,000 permutations. DRC, the Democratic Republic of the Congo.

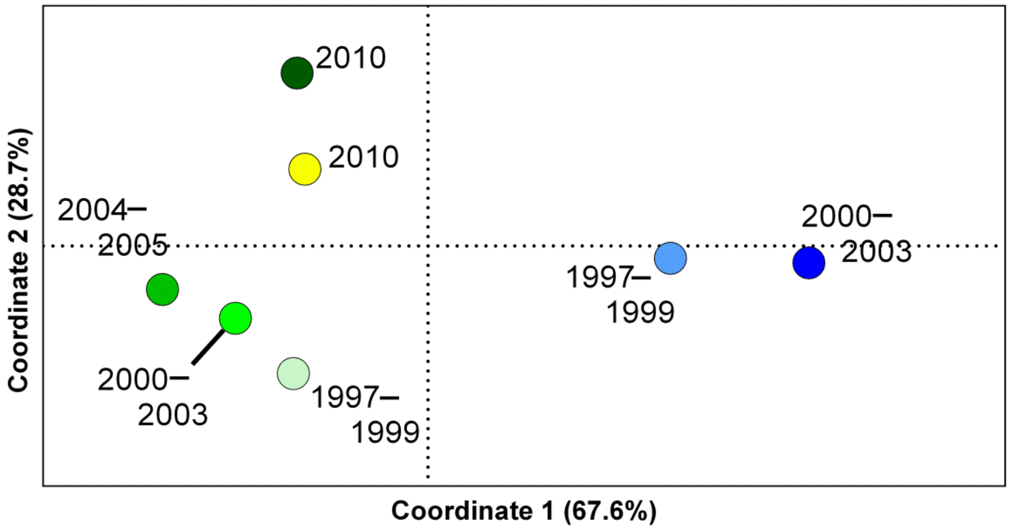

Technical Appendix Figure 1. Genetic relatedness of *Plasmodium falciparum* dihydropteroate synthase (*dhps*) haplotypes from Malawi over time based upon principal coordinates analysis of Φ_{PT} values. Principal coordinates analysis was computed in GenAlEx (*1*) among *dhps* haplotypes binned by year using Φ_{PT} values shown in the Table. Blue dots indicate wild-type SAKA haplotypes, green dots indicate double-mutant S**GE**A haplotypes, and yellow dot indicates S**GEG** haplotypes. Coordinates 1 and 2 accounted for 96.3% of the variance in Φ_{PT} values. The *dhps* haplotypes are defined by amino acids at codons 436, 437, 540, and 581.

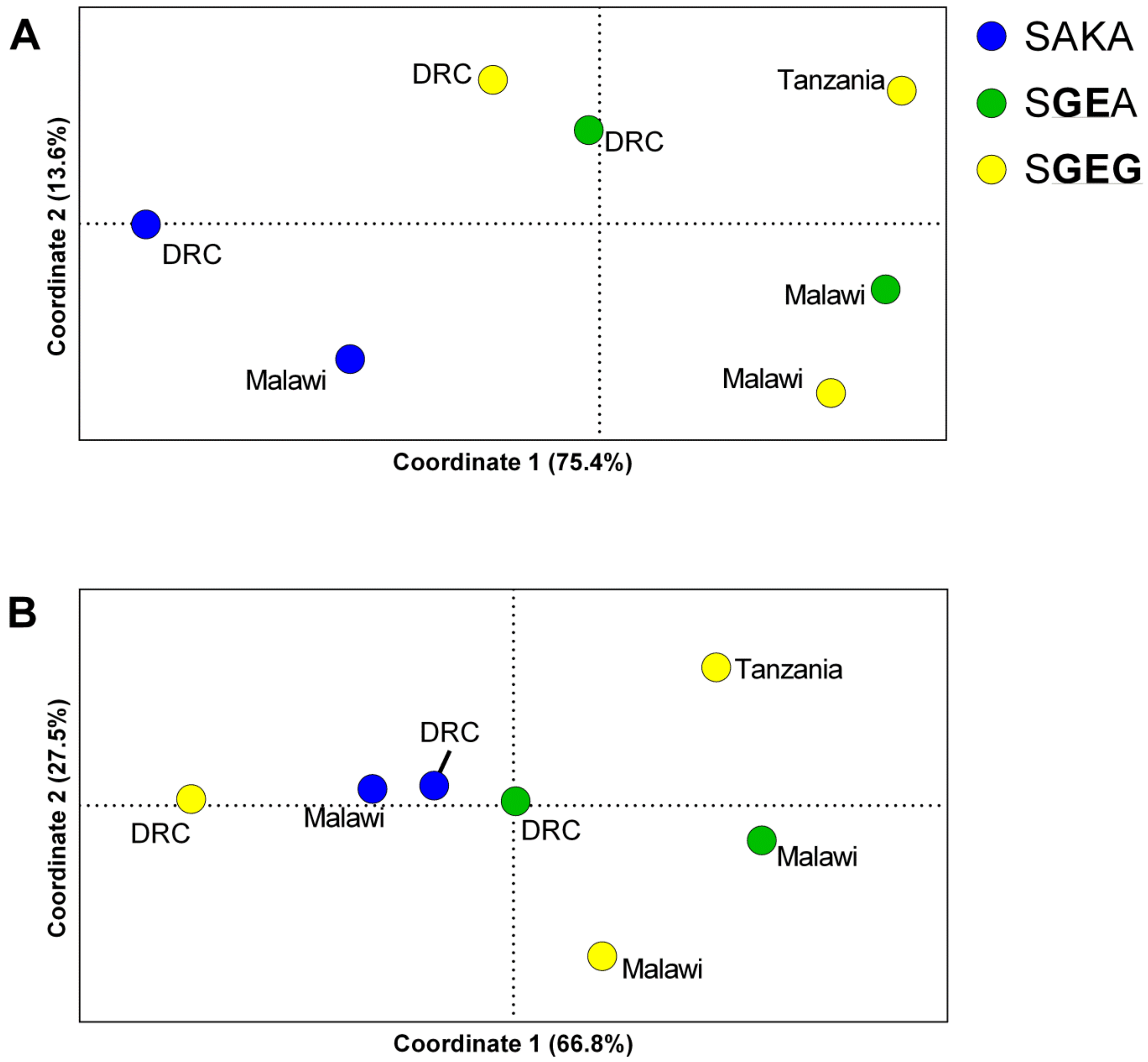

Technical Appendix Figure 2. Principal coordinates analyses of wild-type (SAKA) and mutant (S**GE**A and S**GEG**) *Plasmodium falciparum* dihydropteroate synthase (*dhps*) haplotypes from eastern Africa based upon A) linear genetic distance and B) analysis of molecular variance (Φ_{PT}). Pairwise linear genetic distances and Φ_{PT} were computed with GenAlEx (*2*) among microsatellite profiles of 7 populations of parasites defined by *dhps* haplotype and location: SAKA parasites from Malawi (n = 24) and the Democratic Republic of the Congo (DRC) (n = 53) (blue dots); S**GE**A parasites from Malawi (n = 67) and DRC (n = 17) (green dots); and S**GEG** parasites from DRC (n = 5), Malawi (n = 10), and Tanzania (n = 17) (yellow dots). Pairwise values were input into principal coordinates analyses in GenAlEx, in which coordinates 1 and 2 cumulatively accounted for 89% (A) and 94.3% (B) of the variance. The *dhps* haplotypes are defined by amino acids at codons 436, 437, 540, and 581. Mutant amino acids are indicated in bold.

References

1. Excoffier L, Smouse PE, Quattro JM. Analysis of molecular variance inferred from metric distances among DNA haplotypes: application to human mitochondrial DNA restriction data. Genetics. 1992;131:479–91. PubMed

2. Peakall R, Smouse PE. GenAlEx 6.5: genetic analysis in Excel. Population genetic software for teaching and research–an update. Bioinformatics. 2012;28:2537–9. PubMed http://dx.doi.org/10.1093/bioinformatics/bts460
